# Salivary Biomarkers and Work-Related Stress in Night Shift Workers

**DOI:** 10.3390/ijerph18063184

**Published:** 2021-03-19

**Authors:** Giusi Briguglio, Michele Teodoro, Sebastiano Italia, Francesca Verduci, Manuela Pollicino, Manuela Coco, Annalisa De Vita, Elvira Micali, Angela Alibrandi, Giuseppe Lembo, Chiara Costa, Concettina Fenga

**Affiliations:** 1 Department of Biomedical and Dental Sciences and Morphofunctional Imaging, Occupational Medicine Section, University of Messina, 98125 Messina, Italy; giusi.briguglio@unime.it (G.B.); michele.teodoro@unime.it (M.T.); sebastos87@hotmail.it (S.I.); francesca.verduci92@virgilio.it (F.V.); manuelapollicino@gmail.com (M.P.); coco_manuela@hotmail.it (M.C.); annalisadv90@gmail.com (A.D.V.); concettina.fenga@unime.it (C.F.); 2 Medical Management Staff Unit, University Hospital “Policlinico G. Martino”, University of Messina, 98125 Messina, Italy; elvira.micali@unime.it; 3 Department of Economics, University of Messina, 98122 Messina, Italy; angela.alibrandi@unime.it; 4 Port System Authority of the Strait of Messina, 98100 Messina, Italy; lembo@porto.messina.it; 5 Clinical and Experimental Medicine Department, University of Messina, 98125 Messina, Italy

**Keywords:** shift workers, salivary biomarkers, cortisol, α-amylase, occupational health, work-related stress

## Abstract

Work organization, such as shifts and night work, can interfere with the perception of work-related stress and therefore on the development of pathological conditions. Night shift work, particularly, can have a negative impact on workers’ wellbeing by interfering with the biological sphere. The aim of this study is to evaluate the associations between work activities, shift work effects and stress-related responses in 106 dock workers enrolled in southeast Italy. Dock workers’ tasks consist of complex activities that seemed to affect more sleep quality than work-related stress. An analysis of salivary biomarkers such as cortisol, α-amylase, melatonin and lysozyme was performed along with validated psycho-diagnostic questionnaires. Alpha-amylase showed a significant negative correlation with the effort/reward imbalance score; thus, the measurement of salivary α-amylase is proposed as a sensitive and non-invasive biomarker of work-related stress. This study may provide new insights into developing strategies for the management of night shift work. Salivary biomarkers should be further investigated in the future in order to develop simple and effective tools for the early diagnosis of work-related stress or its outcomes.

## 1. Introduction

Work-related stress is a phenomenon which occurs when work demands are not proportionate to worker resources; it can lead to the onset of harmful physical and emotional reactions. Based on recent studies, environmental factors and some kinds of work organization, such as shifts and night work, can interfere with the perception of work-related stress and therefore on the development of pathological conditions.

Shift work can alter the sleep/wake cycle, producing a disruption of the circadian rhythm of biological function [1,2]. Sleep is the most disturbed function in shift workers, who complain of alterations in terms of its duration and quality with a prevalence ranging from about 10% up to 40% [3,4,5]. As shiftwork has intensified over the years and work-related stress has become a very common phenomenon, researchers have developed over the past decade different types of tools in order to assess employee stress. Existing reporting tools in use to assess the effects of work-related stress include measuring the results of multi-item questionnaires [6,7]. However, subjectivity in the collection of self-administered tests by workers may interfere with the interpretation of questionnaire results. For this reason, researchers have identified scientifically measurable work-related stressors such as biomarkers produced by the human body. Biomarkers are usually assayed in blood, serum or urine samples through long and uncomfortable invasive procedures, and this represents a limitation and an effective obstacle for stress research because of a difficult population recruitment. Actually, biomarker measurement could be performed through less invasive methods such as salivary samples. The use of saliva as a biological matrix for the determination of diagnostic biomarkers is a less invasive fluid collection technique inducing greater compliance in study participants [8].

A high basal salivary cortisol level is predictive of stress resilience [9] and its use has been suggested to monitor work related stress among occupations at risk such as shift workers. However, the development of knowledge on the pathophysiology of organic stress responses has led to the identification of other biomarkers for the quantitative stress assessment, so higher α-amylase levels can be found in conditions of extreme physical activity, anxiety due to high mental effort and also stress due to sudden changes in the external environment, probably linked to the blood concentration of catecholamine norepinephrine (NE) or epinephrine (E) [10,11]. For this reason, many authors have considered α-amylase as a valid and reliable biomarker of the autonomic nervous system activity. Other authors have suggested that salivary lysozyme concentrations are sensitive to stress and could be utilized as a non-invasive biomarker in such studies [12,13] as well as melatonin, a significant biomarker of the circadian clock proposed to differentiate different chronotypes. Melatonin secretion has a cyclical trend during the day, usually high at night (absence of light) and low throughout daylight. It can be measured in blood, urine and saliva samples and it has a tight correlation with night shift work [14,15,16].

While shift work is typical of many service occupations, including hospital workers, data on the effect of different types of work shift rotation and the prevalence of sleep disturbance in relation to shift rotation are still scant [17]. Based on these findings, we conducted an experimental study on a population of dock workers. The aim here is to investigate the inter-relationships of nightwork and work stress with salivary biomarkers such as cortisol, α-amylase, melatonin, and lysozyme to establish whether there are differential effects on these salivary biomarkers by exposure to combinations of work factors.

## 2. Materials and Methods

### 2.1. Study Design and Population

Overall, 106 shift workers operating in the ports of Messina and Milazzo were enrolled for the study in October and November 2019. The subjects, with prior written informed consent, joined according to a health promotion program proposed by the port authority. A multidisciplinary team, made up of medical doctors of the Occupational Medicine Section of the University of Messina supported by a psychologist, illustrated the purpose of the study to the workers, administering validated questionnaires of psycho-diagnostic protocol as well as salivary samples collection kits. These activities were carried out from 7:45 to 12:00 a.m., immediately before the beginning of a work shift. As a criterion for inclusion, a seniority of at least one year was required, while the presence of psychiatric and/or neurological diseases constituted an exclusion criterion, in order to reduce confounding factors. The presence of psychiatric and/or neurological diseases was excluded not only via the self-reported anamnesis but also through the medical examination, conducted by trained medical personnel within the preventive program. A sample of 106 port operators voluntarily agreed to participate in the study. After applying inclusion and exclusion criteria, data related to 105 port operators were used for the subsequent phases of the study.

Trained medical personnel compiled an anamnestic form for each worker, in order to collect and evaluate personal and lifestyle, socio-demographic, health and employment data. Socio-demographic factors included age, gender, marital status and educational level. Health and lifestyle indicators included smoking, alcohol consumption, physical activity, body mass index, systolic and diastolic blood pressure values, coexistence of chronic diseases and recent use of drugs. Occupational factors included work seniority, specific job and duties, work organization and night shift employment, use of personal protective equipment and previous workplace accidents.

### 2.2. Collection of Salivary Fluid Sample

Whole saliva samples were collected in polypropylene 2 mL cryovials and the Saliva Collection Aid (Item No. 5016.02 from Salimetrics, State College, PA, USA), a passive drool specifically designed to improve volume collection and increase participant compliance. This collection device represents the validated gold standard for biological testing of saliva samples. In particular, it avoids localized secretions of specific salivary glands and does not interfere with analytical assessment.

The salivary sample was collected in the morning, two hours after awakening and before the psycho-diagnostic tests were administered. None of the subjects were requested to collect saliva after a night shift. According to the best practices in saliva sample collection, participants were asked to fill the vial following a specific procedure aiming to avoid alteration of the sample composition or specimen contamination with substances that could interfere with the immunoassay, such as avoiding foods with high sugar or acidity, or high caffeine content, immediately before sample collection; documenting consumption of alcohol, caffeine, nicotine, and prescription/over-the-counter medications within the prior 12 h; not eating a major meal within 60 min of sample collection; rinsing mouth with water to remove food residue and waiting at least 10 min after rinsing to avoid sample dilution before collecting saliva. Moreover, the use of unstimulated, whole saliva collected by the passive drool technique allows to maintain consistency in sample composition and flowrate.

In addition, each worker was given a second kit for taking an additional salivary sample at their home at 10:00 p.m. for cortisol determination, collected by themselves after having been adequately instructed.

All samples were rapidly stored at −20 °C until analysis.

### 2.3. Determination of Salivary Enzymes on Collected Samples

On the salivary samples collected in the morning the assay of cortisol, melatonin, α-amylase and lysozyme were performed. Only the evening cortisol level was measured on salivary samples collected at 10:00 p.m. Salivary α-amylase, cortisol and melatonin were analyzed using, respectively, the salivary α-amylase kinetic enzyme assay kit (Item No. 1-1902, Salimetrics, State College, PA, USA), the cortisol enzyme immunoassay kit (Item No. 1-3102, Salimetrics, State College, PA, USA) and the melatonin enzyme immunoassay kit (Item No. 1-3402, Salimetrics, State College, PA, USA). The lysozyme assay was performed using the ELISA kit from the Innovative Research, Inc. manufacturer (Innovative Research, Novi, MI, USA).

Salivary levels on each analyte were expressed as concentration or enzyme activity per ml. Though the concentration of some hydrophilic analytes could be affected by the flow rate, results were not normalized for this parameter. Thanks to the use of the passive drool device for sample collection, this effect should be negligible. Moreover, the measurement of the flow rate for the evening sample collection would not have been reliable because of the lack of a supervisor control.

### 2.4. Psycho-Diagnostic Protocol

The validated questionnaires and psycho-diagnostic tests required by the protocol were administered to individuals by trained medical personnel, before the start of the daytime work shift and after having filled in the anamnestic data collection form.

#### 2.4.1. Effort/Reward Imbalance

Stress experience was assessed using the validated short Italian version (16 items) of the effort/reward imbalance (ERI) questionnaire, one of the most used theoretical models for the purpose of evaluating psycho-social factors in the workplace [18]. It is a standardized questionnaire containing items concerning effort (both psychological and physical), reward (obtained in exchange for efforts made at work and including satisfactory salary, appreciation and career advancement) and over-commitment (individual personality component). The effort scale is measured by three items (ERI1–ERI3), the seven-item reward scale (ERI4–ERI10) and the overcommitment scale containing six items (ERI11–ERI16), are proposed in the form of a Likert scale with four possible answers (strongly disagree, disagree, agree, strongly agree). Scores are obtained following the Siegrist algorithm and, following normalization, the effort/reward ratio is calculated. In the case of an effort/reward (E/R) ratio equal to 1, each individual effort corresponds to a reward; for an E/R ratio <1, there are fewer efforts for each reward; for an E/R ratio >1, the subject reports more efforts for each reward.

#### 2.4.2. Pittsburg Sleep Quality Index

Sleep quality is measured by the Italian version of the Pittsburg Sleep Quality Index (PSQI) [19]. It is a questionnaire that evaluates the quality of sleep over a one-month time interval. The PSQI consists of 19 items and evaluates seven different aspects of sleep and one composite score: the subjective quality of sleep, the time taken to fall asleep, the duration of sleep, habitual sleep performance (for example, the percentage of time spent asleep in bed), presence of sleep disturbances, use of hypno-inducing drugs, and dysfunction in daily life. Each of the seven aspects can be scored on a 0–3 scale. The overall PSQI score is then calculated by adding up the seven component scores on a scale ranging 0–21, with a maximum score of 63. High scores indicate greater sleep impairment (≤5 indicates good sleep quality; >5 indicates poor sleep quality).

### 2.5. Statistical Analysis

Categorical variables were expressed as absolute and percentage frequencies; numerical variables such as mean, standard deviation, minimum and maximum. The non-parametric approach was used for the analysis of the data since the variables examined are represented by scores obtained following the administration of questionnaires. The Spearman correlation test was applied in order to assess the existence of possible interdependent relationships between the different questionnaires and between the individual biomarkers. Furthermore, the possible association between questionnaire items, socio-demographic variables, work organization variables, lifestyle variables and health factors with salivary biomarkers was investigated. Statistical analyzes were performed with SPSS for Windows software version 22 (IBM, Armonk, NY, USA), considering a significance level of 5%.

## 3. Results

### 3.1. Socio-Demographic Factors

Table 1 reports the sociodemographic characteristics and lifestyle of enrolled subjects.

Of a total of 105 port operators included in the study (104 males and 1 female), 52 individuals (49.52%) were within the range of 26 to 45 years old and 53 (50.48%) were over 46 years old. About 78.09% of subjects (82) were married, with a percentage of 74.28% (78 subjects) having children. As regards the degree of education, 45.71% had a diploma, against 54.29% of subjects with less than eight years of schooling.

In addition, subjects were divided on the basis of occupational seniority (≤20 years and >20 years), with a percentage of 31.43% (33 workers) and 68.57% (72 workers), respectively. All the enrolled subjects were shift workers, but 75.24% currently worked on night shifts and only 26.67% (28 individuals) reported having had occupational and/or extra-occupational injuries.

Dealing with the habit of alcohol consumption and cigarette smoking, it was shown that 52.38% (55 workers) consume alcoholic beverages and only 40% (42 subjects) are smokers. 

Concerning health status, 67 subjects (63.81%) were overweight or obese (BMI > 25). With regard to blood pressure values, the results showed that 26.67% of workers (28 individuals) had systolic blood pressure values ≥ 140 mmHg, while 29.52% (31 subjects) had diastolic blood pressure values ≥ 90 mmHg.

### 3.2. Stress and Sleep Evaluation

#### 3.2.1. Effort/Reward Imbalance (ERI) Questionnaire

Table 2 shows the results of the effort/reward imbalance test. The test showed a low mean effort level (4.48 on a 3–12 scale) and a high mean reward (22.0 on a 7–28 range). All subjects showed an E/R ratio < 1, with 0.106 average, suggesting that the reward obtained was considered adequate for effort. This finding was confirmed by a scarcely perceived overcommitment (9.38 ± 3.76, mean ± *SD* on a 6–24 range).

#### 3.2.2. Pittsburgh Sleep Quality Index (PSQI)

The results of the Pittsburgh sleep quality index (PSQI), reported in Table 2, revealed an average overall score of 3.96, within the range of 0–13. Furthermore, 70.48% of subjects showed values < 5, suggestive of good sleep quality, while mild sleep alterations were reported in 29.52%.

### 3.3. Salivary Biomarkers

Saliva samples collected in the morning were suitable for analysis in 83 subjects, showing an average morning cortisol value of 0.192 µg/dL (range 0.010–1.02 µg/dL); a mean melatonin value of 6.74 pg/mL (range 0–52.5 pg/mL); mean α-amylase level of 100 U/mL (ranging 0.328–500 U/mL); an average lysozyme value of 3.42 µg/dL (1.23–5.25 µg/dL). Salivary biomarkers concentrations are summarized in Table 3.

Among the 83 port operators, only 51 subjects also delivered the saliva samples collected in the evening at their home. In these subjects, an average evening cortisol value of 0.078 µg/dL was shown, with a range between 0 and 0.387 µg/dL.

No subject showed morning cortisol values higher than the reference range in healthy populations, as shown in Table 4. Of the sample of 51 dock workers who completed the collection of all samples, 50 subjects (two aged between 21 and 30 years, 29 between 31 and 50 years, 19 between 51 and 64 years) showed evening cortisol values including in the reference range in control populations; only three subjects showed an evening cortisol value above the reference range as summarized in Table 4.

Among 83 subjects, eight showed melatonin values within the reference range; on the other hand, 64 subjects and 11 subjects showed melatonin values respectively lower and higher than the reference range, as shown in Table 4.

The α-amylase assay showed values within the reference range in 68 subjects; values lower than the reference range were found in nine subjects and six subjects showed higher α-amylase levels, as shown in Table 4.

All subjects showed lysozyme values within the reference range, as shown in Table 4.

### 3.4. Statistical Association

**Figure 1:**
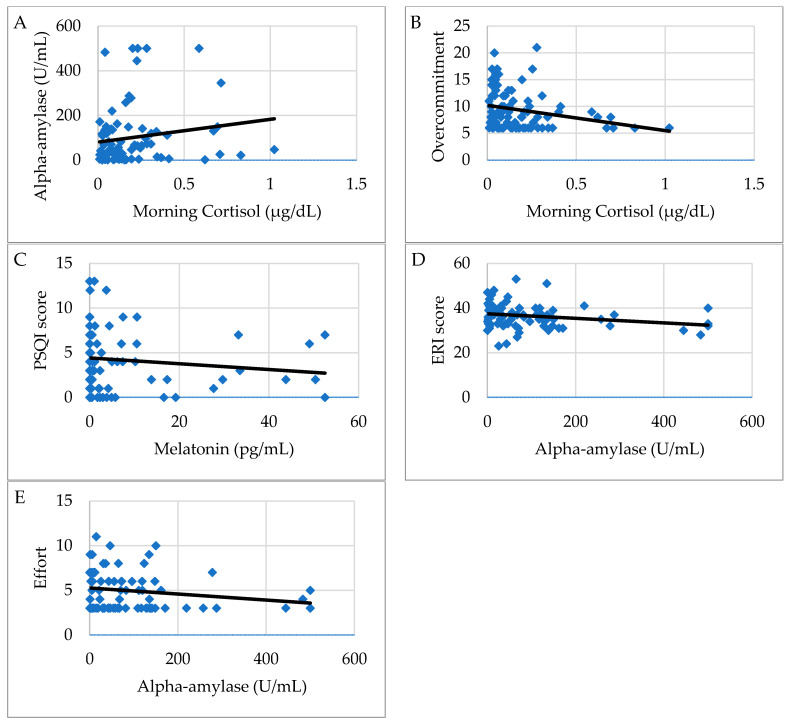
(**A**–**E**). Graphic representation of correlations between questionnaire scores and salivary biomarkers in dock workers. Figure shows the association of morning cortisol levels with α-amylase (**A**) and overcommitment scale of ERI (**B**), of salivary melatonin with Pittsburgh sleep quality index (PSQI) score (**C**), of α-amylase concentration with Effort reward imbalance (ERI) total score (**D**) and effort scale (**E**).

Morning cortisol showed a statistically significant positive correlation with α-amylase values (r = 0.219, *p* = 0.047) as shown in Figure 1A; a negative correlation was found with the evening cortisol values (r = −0.314, *p* = 0.025) and with the Overcommitment scale of the ERI questionnaire (r = −0.264, *p* = 0.017) (Figure 1B).

Evening cortisol showed a negative correlation with body mass index (r = −0.300, *p* = 0.032) and with age (r = −0.292, *p* = 0.038), but a positive correlation with educational level (r = 0.302, *p* = 0.031).

Melatonin showed a positive correlation with the organization of night shift work (r = 0.345, *p* = 0.001); a negative correlation was found with the PSQI scores (r = −0.254, *p* = 0.021) as shown in Figure 1C.

Alpha-amylase showed a negative correlation with the Siegrist ERI scores (r= −0.273, *p* = 0.013) and principally with the Effort (E) scores (r = −0.226, *p* = 0.042) as shown respectively in Figure 1D,E.

Lysozyme did not show statistically significant correlations with the values of the other salivary biomarkers, nor with socio-demographic factors, nor with the scores achieved in the psycho-diagnostic protocol tests.

PSQI scores presented a positive correlation with occupational seniority (r = 0.213, *p* = 0.029), with age (r = 0.296, *p* = 0.002), with the overcommitment scores of the Siegrist ERI questionnaire (r = 0.322, *p* = 0.001).

The scores of effort (E) of the ERI questionnaire showed a positive correlation with educational level (r = 0.251, *p* = 0.010); on the other hand, a negative correlation was found with body mass index (r = −0.195, *p* = 0.048). The E/R ratio presented a positive correlation with schooling degree (r = 0.198, *p* = 0.043). The overcommitment (O) scores of the Siegrist ERI questionnaire showed a negative correlation with the organization of night shift work (r = −0.208, *p* = 0.034).

## 4. Discussion

Reduced sleeping time, caused by night shift work or irregular working schedule, can lead to chronic fatigue and reduced levels of attention and vigilance, with a possible increase in occupational injuries. This population of dock employees worked on night shift at least once a week and followed irregular working patterns, but not all of them suffered from sleep disorders as evaluated by PSQI. Considering a score ≥ 5 as suggestive of sleep disorders, 39/105 (37.1%) subjects reported sleep dysregulation, which is in agreement with existing literature [4]; importantly, the majority had a mild impairment of sleep quality, whereas only 4/105 (3.8%) presented a PSQI global score > 10. 

Moreover, PSQI was negatively related to melatonin, which presented low salivary levels in most subjects, in line with other similar studies that have used this biomarker in shift workers [15,16]. This is due to the sampling time, but probably also to the alteration of the normal circadian rhythm [20]. Though, if in one hand the majority of workers in our sample showed low salivary melatonin levels, on the other hand, a higher level of melatonin was associated with the organization of night shift work. This association, in apparent discordance with previous results, might be explained as a circadian rhythm disorder [21,22]. In fact, lower melatonin levels have been reported in night workers, due to increased light exposure and desynchronization of the sleep/wake cycle; the timing of melatonin production can be altered, and a single morning sampling might undervalue the whole melatonin production [23].

Dock workers’ tasks consist of complex activities. They carry out their job in extremely difficult, potentially dangerous conditions requiring physical and mental engagement, including operating by the sea in adverse weather conditions, prompt availability and unpredictable work assignments, a high level of effort and commitment, requiring high flexibility both for workers and their families. This prolonged condition of intense strain in both the personal and working sphere can be overcharged by ageing and by the onset of health disorders including stress.

Unexpectedly, in this workforce, a low stress level was found, as assessed by Siegrist’s ERI questionnaire. In fact, all subjects showed an E/R ratio < 1, suggesting that a favorable balance was found between work demands, individual resources and the reward obtained. However, results indicate a trend towards increased overcommitment when compared to other ERI subscales, suggesting a propensity for high involvement in work.

In previous research, the ERI questionnaire has been related to salivary cortisol in different working categories [24,25,26,27,28]. Nonetheless, we did not find any studies investigating the relation between ERI and cortisol levels in night shift workers.

All study participants presented salivary morning cortisol levels in the normal range, in agreement with results of the stress-related questionnaire. Morning cortisol levels were negatively associated with overcommitment, but an explanation of this outcome is not straightforward. We can hypothesize that, given the tasks of this employed population and the work organization, overcommitment does not necessarily reflect a negative outcome, but it could represent a protective factor against work-related pressure, hence resulting in a negative association with cortisol that, conversely, mirrors high stress levels.

Also, data available from other research are discordant; in fact, whilst in several studies overcommitment is positively associated with cortisol levels [24,26], others have not found any relation [25,27].

Overcommitment was shown to be positively associated also with sleep disturbances, in agreement with existing research suggesting that high overcommitment can be a risk factor for reduced sleep quality [29]. 

Finally, morning cortisol and α-amylase presented an overlapping trend, showing a positive association. However, morning cortisol was not altered in any of the subjects in our sample; conversely, α-amylase showed variations in 10% of subjects, thus suggesting a greater sensitivity in disclosing an alteration compared to cortisol [30]. 

Additionally, evening cortisol levels were observed to be slightly higher than reference values in only three subjects (6%), but no relevant association with work, health or sociodemographic factors was found.

While the cortisol diurnal profile has been well investigated and described to be altered in several pathological conditions, similar research on α-amylase levels is only just beginning. Data in the literature report that a-amylase activity is a salivary biomarker with a typical diurnal pattern, with an opposite trend compared to cortisol [31]. The diurnal profile of α-amylase activity usually observes a dip after awakening and rising levels throughout the day. Salivary α-amylase has been proposed as a sensitive biomarker for stress-related changes in the body that reflects sympathetic nervous system activity. Moreover, some studies are evaluating the validity and reliability of this parameter. Indeed, under this premise, our study hypothesizes that salivary α-amylase may be sensitive to stress-related alterations. However, this field of research is still in its early stages and the use of salivary α-amylase as an indicator of dysregulation of the sympathetic-adrenomedullary system is promising.

Our results showed that 80% of enrolled workers presented salivary an α-amylase level in the normal range according with the results of the stress-related questionnaire. Moreover, α-amylase levels were negatively associated with ERI total score; in particular, α-amylase was associated with the effort subscale. Therefore, a work activity characterized by greater effort led to lower levels of α-amylase; these results were contrary to our expectation. Indeed, most recent literature data relate increased levels of α-amylase to various stress factors, such as cardiovascular risk [32] and anxiety [33,34,35]. These contrasting results could be partly explained by the time of collection; in fact, the morning collection of the salivary sample overlaps as a time span with the negative post-awakening peak.

In contrast to α-amylase and cortisol, the available data on salivary lysozyme are scarce. The results of our study, aiming to investigate a possible interrelationship between work-related stress and salivary lysozyme, did not show alterations when compared to the reference values, in agreement with other authors [36].

Therefore, workers enrolled in this study did not show a high level of perceived stress in relation to occupational risk factors, but also resulted to be an overall healthy male population (only one woman was included) with a homogeneous working pattern. Half of them were >46 years old and almost 70% had been employed in the same job for more than 20 years without reporting occupational injuries; no recent use of medication and chronic diseases were reported; in particular, no subjects with psychiatric illness were enrolled. Alcohol consumption and cigarette smoking, which are further factors potentially influencing individual differences in stress-related salivary biomarkers, showed no effect on the results.

Notwithstanding the peculiar characteristics of this working population, the determination of salivary α-amylase levels could be proposed as a sensitive non-invasive biomarker of work-related stress. Conversely, lysozyme did not show significant variability in salivary samples from this population, probably confirming the absence of stress indicated by the ERI questionnaire. 

Although complex skills are required of dock workers in critical situations (such as the management of unforeseen events), in our population, no alteration of the psycho-emotional state of the workers was detected in response to night shifts, in contrast to other working categories [37].

This is one of the few studies investigating the impact of night shift work and stress on dock workers, a peculiar working population; similar studies generally focus on healthcare workers. This study also presents some limitations, such as the small sample size; moreover, though we found various statistically significant associations, the study design does not allow us to establish any causation, so the direction of some relationships is not known. Furthermore, it would be interesting to compare working populations belonging to different job backgrounds in order to evaluate the usefulness of different schedules of night shifts and stress impact from a more inclusive point of view.

## 5. Conclusions

Night shift work can trigger a desynchronization of the circadian rhythm, interfering with psychophysical sphere. Workers may experience a different behavior in response to these stressors, as stress is a physiological reaction adaptive to the environment and to different life situations. Nonetheless, a well-organized turnaround could help to preserve the psycho-physical health of workers.

Dock workers’ tasks consist of complex activities that seem to affect sleep quality more than work-related stress. In fact, while about 40% of subjects suffered from sleep disturbances, none of the participants reported an effort/reward imbalance. Moreover, the results of the two main stress salivary biomarkers—cortisol and α-amylase—were also in accordance to ERI questionnaire.

One of the key points of this research is in support of the employment of salivary stress biomarkers in clinical practice. A primary goal would be to screen workers performing shift work; in light of this, using validated salivary biomarkers as indicators of work-related stress disorders could support the implementation of these strategies. Administrators should guarantee ergonomic shift work, promoting sleep hygiene programs to improve workers’ wellbeing.

In conclusion, adopting rigorous methodological recommendations to avoid factors that influence and add variance to stress biomarker measurement, this investigation suggests that the use of saliva as a diagnostic fluid could support subjects in improving their coping strategies against difficult life events, through non-pharmacological approaches.

## Figures and Tables

**Table 1:** Sociodemographic characteristics and lifestyle of study population.

Variables	Categories	*N* = 105	%
Sociodemographic data			
Age			
	26–45	52	49.52
	>45	53	50.48
Gender			
	Male	104	99.04
	Female	1	0.96
Marital status			
	Married	82	78.09
	Not married	23	21.91
Children			
	Yes	78	74.28
	No	27	25.72
Degree of education			
	Secondary school or higher	48	45.71
	Primary school or lower	57	54.29
Health and lifestyle variables			
Body mass index			
	18–25	38	36.19
	>25	67	63.81
Blood pressure values			
	Systolic ≥ 140 mmHg	28	26.67
	Diastolic ≥ 90 mmHg	31	29.52
Smoking status			
	Smoker	42	40.00
	Non-smoker	43	40.95
	Ex-smoker	20	19.05
Alcohol consumption			
	Yes	55	52.38
	No	50	47.62
Work-related factors			
Occupational seniority			
	≤20 years	33	31.43
	>20 years	72	68.57
Night shifts			
	Yes	79	75.24
	No	26	24.76
Occupational and/or extra-occupational injuries			
	Yes	28	26.67
	No	72	73.33

**Table 2:** Results of the Effort/Reward Imbalance Test and Pittsburgh Sleep Quality Index.

Psycho-Diagnostic Tools	Mean ± *SD*	Minimum	Maximum
ERI ^1^ (TOTAL SCORE)	36.20 ± 5.54	22	53
EFFORT	4.48 ± 2.22	3	12
REWARD	22.00 ± 5.11	7	28
E/R ^2^	0.106 ± 0.072	0.046	0.396
OVERCOMMITMENT	9.38 ± 3.76	6	21
PSQI ^3^	3.96 ± 3.13	0	13

^1^ ERI: effort reward imbalance; ^2^ E/R: effort/reward ratio; ^3^ PSQI: Pittsburgh sleep quality index.

**Table 3:** Levels of salivary biomarkers.

Salivary Biomarkers	Mean ± *SD*	Minimum	Maximum
MORNING CORTISOL (µG/DL)	0.192 ± 0.207	0.010	1.02
EVENING CORTISOL (µG/DL)	0.078 ± 0.114	0.000	0.387
MELATONIN (PG/ML)	6.74 ± 13.20	0.000	52.50
ALPHA-AMYLASE (U/ML)	100.0 ± 131.2	0.328	500
LYSOZYME (µG/ML)	3.42 ± 1.34	1.23	5.25

**Table 4:** Sample frequencies in salivary biomarkers range values.

Salivary Biomarkers	Reference to Limit Values	*N*	%
Morning cortisol	(µg/dL)		
Age 21–30	<0.743 *	5	6.02
	>0.743	0	0
Age 31–50	<1.551 *	50	60.24
	>1.551	0	0
Age 51–70	<0.812 *	28	33.74
	>0.812	0	0
Evening cortisol	(µg/dL)		
Age 21–30	<0.308 *	2	3.92
	>0.308	0	0
Age 31–50	<0.359 *	29	56.86
	>0.359	1	1.96
Age 51–70	<0.228 *	17	33.34
	>0.228	2	3.92
Melatonin	(pg/mL)		
	<6.7	64	77.11
	6.7–17.1 *	8	9.64
	>17.1	11	13.25
Alpha-Amylase	(U/mL)		
	<3.1	9	7.84
	3.1–423.1 *	68	81.83
	>423.1	6	7.23
Lysozyme	(µg/mL)		
	<1	0	0
	1–11 *	83	100
	>11	0	0

* Normal cut-off values.

## Data Availability

The datasets used and/or analyzed during the present study are available from the corresponding author on reasonable request.
